# Notes and Comments

**Published:** 1922-01

**Authors:** 


THE
### HOSPITAL and HEALTH REVIEW

### NOTES AND COMMENTS.

LAST month we commented on the fact that there seems to be a case for the revision of the Public Health Acts. These Acts deal with many different subjects, and there are many amending Acts, some of which are voluntary, in that they need not be adopted in an area. In two neighbouring districts entirely different standards of sanitary legislation may prevail—for example, a drain may be a sewer on one side of the street, and a private drain on the other. If the Acts on Health and Housing were not so difficult to interpret and to administer they might almost be regarded as humorous ; as it is, they give much worry to and waste much of the time of the officers of Local Authorities. It would surely not be difficult to codify the existing Acts and incorporate into the n w legislation several greatly needed amendments. Probably the best method of undertaking this reform would be for a series of Bills to be drafted, each dealing with a department of public health activity. For example, a Public Health (Food) Bill might codify, revise and amend all matters connected with food and health ; a Public Health (Housing) Bill might similarly deal with all matters relating to housing, such as the abatement of nuisances, houses let in lodgings, common lodging houses, and housing schemes. A Public Health (Infection Diseases) Bill would co-ordinate similarly the existing laws on this subject. A Public Health (General Provisions) Bill might include such subjects as could not well be included in any other enactment. There are very many Local Authorities in England and Wales who have strengthened their public health services by means of useful provisions in private Acts of Parliament, and it would be most desirable for the good of the country as a whole to adopt in the general revision such clauses as have been found of value locally. Again, we might take some hints from the public health legislation of some of our Colonies, such, for example, as Australia and New Zealand. In the lean years that are before us we might well spend some time in setting our house in order, and in consolidating the basis from which public health is administered in this country. It would not cost money to review and revise our ancient laws, and it would be of a real advantage both immediately and in the future.

IT may be interesting to give a concrete example of the difficulties under which sanitary reforms are carried out to-day. We may imagine that a house-drain is choked, that it cannot readily be cleared and that the sewage is beginning to flow over the kitchen and scullery floor. The attention of the Health Department is called to this gross condition, and probably the medical officer of health visits the house. The landlord or responsible person is told at once that he must do the work necessary to make matters right ; but he takes no notice of the request. Three weeks or a month may pass before the next meeting of the Health Committee, and meanwhile the overflow in the scullery is an inch deep. At the Health Committee a resolution is passed to serve a statutory notice upon the landlord, and that resolution is approved at the Town Council, say, a fortnight later. The notice is served on the landlord, who puts it in the fire, and there are by now two inches of sewage on the scullery floor. Yet another month has to elapse before the next meeting of the Town Council, which passes a resolution to prosecute the landlord, and meanwhile the scullery is uninhabitable and definitely a menace to health. A week later the owner is duly prosecuted ; but before the case comes on at the Police Court he has probably done the necessary work, and receives, therefore, from the magistrate only a nominal penalty. Altogether from the time when the drain became choked to the time when it was cleared was, at any rate, a couple of months—which seems incredible in a civilised country. This is not an exaggerated case, and it shows the difficulties under which the public health services labour. To give summary powers to a health department that they may deal with cases such as this, seems to be eminently desirable.

**NOTES AND COMMENTS**—(*cont.*).

THE Reform of the Poor Law is of course a hardy annual, and no Government has yet been discovered that is able or willing to deal with the problem. There are very few people, except possibly Clerks to Guardians, who would not welcome the redistribution among other authorities of our present poor law system. Commission after Commission has sat to consider the Poor Law, and the result of it all is that in practice we still have the mixed workhouse. Certainly in many places, perhaps by now in all, the children have been removed from the rooms which are peopled with the aged, the diseased, the imbeciles and mentally defectives, and the girl who is waiting to give birth to her illegitimate child. But though, tardily enough, the children have been taken away, the remaining mixture is loathsome and hateful. No decent old woman will willingly go to the workhouse; no young girl in trouble would stay in the place, if she had anywhere else to go while she is waiting. One of our reviewers wrote recently: "It is not necessary to overstate a good case, nor to protest too much against an evil"; so we need not further insist on the undesirability of the mixed workhouse. Of course to-day we have no paupers—they are poor persons; and our infirmaries have become hospitals. But the mere alteration of a few names will not reform the system. It may well be thought that the revision of our system of Poor Law administration is too large and too expensive a matter for us to undertake at this time; but a little might be done. Vaccination, for example, might be handed over to the Health Authorities; and the care of the woman with the illegitimate child might without much expense devolve upon our existing Maternity and Child-welfare Committees. In time, in a decade or so, by such a process of attrition, the existing evils would be remedied, and the reformation would have been accomplished so quietly and unobtrusively that we should hardly have been aware of it.

---

WE publish in this number an interesting article by Dr. A. R. Friel on the treatment of discharging ears by means of ionisation with a solution of a zinc salt. It may be argued by some that the treatment of this condition is not a matter of public health interest, but rather one for the clinician; but we think otherwise. This distressing and dangerous condition, so common in children, tends, if it remains untreated, to permanent injury and to deafness of great or small extent. If the ear discharge is treated early and thoroughly, the condition is eminently curable, and the child is saved from more remote but greater danger. The same is true of many other, so-called minor, defects; in themselves and for the moment they are no doubt trifling, but like the small hole in the stocking they become in time incurable. The slight defect in eyesight if corrected early will not lead to subsequent diminution of vision; and the single carious tooth when filled or extracted will not cause general oral sepsis. It may be true, as a correspondent told us last week, that only 5 per cent. of tonsils are actually diseased—it is a pity that this figure is so high. Preventive medicine should not wait for definite disease; it should offer treatment at the earliest possible moment before the disease becomes of importance. If all children had their enlarged tonsils removed as soon as this enlargement was observed there would be less than 5 per cent. of "diseased" tonsils. To wait until the streptococcus or the tubercle bacillus has caused actual signs of disease is to wait too long We welcome, therefore, this article by Dr. Friel, since it seems to offer a solution of the difficult problem of providing early and successful treatment for this distressing and dangerous condition.

---

THE Pound for Pound principle has been adopted by the Government as the basis for the issue of their grants to the voluntary hospitals. Our hospitals, apart from those appropriated to the treatment of infection diseases, are not State or rate-aided institutions. They are private enterprises working for the public benefit, and they exist upon their endowments and upon the voluntary contributions of the public. An ordinary grant made by the State might have turned them into State institutions; but a wise policy has averted that necessity. The State, in effect, has said to the hospitals: "I will give you half a million pounds if half a million other persons will each give you one pound." We often hear of voluntary subscribers making similar conditions regarding their gifts; for example, a wealthy silk merchant gives a thousand pounds to the Society for the Welfare of Silkworms on the condition that so many other subscribers can be found to put down such and such amounts. It is a common way of stimulating public and private interest; and the State, by becoming a voluntary subscriber to the hospitals, has adopted a well-known and admirable method of procedure. A definite grant in aid of hospitals would have necessitated State control, in the same way as other grants in aid give control of the State over the school clinics, for example, or the sanatoria or maternity and child welfare centres. The voluntary hospitals did not wish to be State institutions, although certainly they needed aid either from the Government or from anyone with money; and the present proposal of "a pound for a pound" seems the only possible way out of the difficulty involved in giving public money without demanding public control. We congratulate the Ministry of Health on having discovered this easy way of escape from a political difficulty, and we congratulate also the hospitals who will receive their money on such easy conditions. Of course, there will be some grumblers; but only among those few hospitals which have been indolent in stimulating public opinion in their districts, or have been dilatory in their work of propaganda. These few will no doubt take refuge behind the fact that the pound for pound principle was not mentioned in the Cave Report. That is neither here nor there. Quite a large number of things regarding propaganda and the collection of money were not mentioned in the Cave Report. We think it is likely that those hospitals which quote the Report against the pound for pound principle are just those hospitals

**NOTES AND COMMENTS**—(*cont.*).

which would be the last to agree with and to carry out the really important recommendation contained in the Report.

---

IN a letter to the "Times," the Minister of Health, Sir Alfred Mond, has clearly outlined the policy of the Government regarding the subsidy to the voluntary hospitals. Especially he makes the point that an unconditional grant from the Government, rather than aiding the hospitals, would be the first step to their disintegration or absorption by the State. The public, seeing the giving of an unconditional grant, would say, "Well, we need not give to the hospitals any longer—the Ministry of Health will look after them. Why should we pay twice—once in taxes, which are heavy enough, and once in contributions of a voluntary nature? No more subscriptions from us!" The vicious circle would be started, and in a very few years there would be no voluntary contributions at all. All of us must have seen this happening in other spheres of life. Ten years ago, for example, a maternity centre was started by voluntary subscription and staffed by voluntary workers. With the coming of the State grant and the assistance of the ratepayers, the voluntary contribution disappeared. We consider that the Ministry of Health has been wise in its policy of grants to the voluntary hospitals, and we welcome, therefore, the pronouncement of Sir Alfred Mond, which we print, without further comment, on page 106.

---

THE humble louse, whose annoying activities have been directly responsible for millions of cases of typhus and thousands of deaths in post-war Europe, is the subject of an interesting report issued by American Red Cross headquarters in Paris. The report describes the anti-typhus work done by the Red Cross during a very severe epidemic of typhus at Cattaro, Dalmatia, resulting in nearly 40 per cent. of deaths among Russian refugee patients there. The Red Cross sent a special corps of typhus specialists to the scene, and the epidemic was finally stamped out. Part of the report is devoted to describing the precautions taken to protect doctors and nurses engaged in the anti-typhus work. Protection of the personnel in the hospitals depends mainly on the efficiency of the disinfecting squad, writes Dr. Ransom. Typhus is not directly infectious—it can only be carried by the medium of the louse. Therefore, if patients are properly disinfected, there is absolutely no danger to the hospital personnel. In this respect, typhus is similar to malaria. There is no danger from a patient with malaria, nor is there any danger from a patient with typhus. At our stations, every refugee was put through a delouser. All the hair was clipped close, and the patient was scrubbed thoroughly with soap and hot running water. The body was anointed with kerosene or coal-oil. Clean pyjamas were placed on the patient, and he was wrapped in a clean blanket and transferred to his bed in the hospital. His clothes were put through a pressure steam steriliser, and furs were put through a sulphur bath. In this way only louse-free patients arrived at the hospital. Any person finding a louse on a patient or on the clothing of the personnel was required to report this immediately to the physician in charge of the hospital, or to the chief nurse, at which time special steps were taken to discover from what source the louse came. It is seldom necessary for physicians or nurses to expose themselves by handling louse-infected patients and articles of clothing, as there are always present persons who have had typhus who can handle typhus patients and their clothing. The work of the physician and the nurse should be directing, and not handling, patients or articles of clothing. Too many over-enthusiastic persons think they must demonstrate the fact that they are not afraid of typhus or of work. These people are penny wise and pound foolish. They are sure to be infected sooner or later, and then their work must be done by someone else.

---

STEPS are being taken to organise a Hospital Committee for the whole of Yorkshire to supervise and co-ordinate the working of the hospitals in that county. The Hospital Commission of the Ministry of Health proposed that there should be a Committee set up for each of the following districts: Leeds, Bradford, Sheffield, and the West, East and North Riding Administrative Counties. Apparently, however, the local hospital authorities feel that they will be less trammelled by a Committee for the whole county rather than by a number of local committees; and, that, working under a County Committee, there will be less danger of interference in matters of domestic detail. We regret their decision for two reasons: Firstly, it shows evidence of unnecessary apprehension regarding the functions of the proposed Committees, a fear which, we believe, to be entirely unjustified; and secondly, this proposed Committee for the whole county cannot obviously be closely associated with the various local authorities, who are daily making more and more use of the voluntary hospitals in their areas. We believe that there ought to be the closest and most cordial co-operation between the various health authorities and the voluntary hospitals which serve the districts. This co-operation can be most easily brought into being by the formation of committees, as recommended by the Cave Committee, on which the local health authorities are represented.

---

THE temporary discontinuation of the "Charity Organisation Review" leaves a gap in institutional journalism. But, thanks largely to the paper's own work, the gap is less than it would have been some years ago. What may be termed social work has now many journals, none the less valuable for being local ones. While it is true that we have never seen precisely eye to eye with the "C.O.S." and the aims which inspired it, it is also true that the old philanthropy was none the worse for a critic in whose eye inspection and the pigeon-hole loomed largely. We hope that in time the REVIEW will resume publication and continue to preach the pure milk of the word in its pursuit of organisation and efficiency.

**NOTES AND COMMENTS**—(*cont.*).

AT a recent meeting of the Yorkshire Federation for Maternity and Child Welfare, which was held at the Town Hall, Sheffield, the Medical Officer of Health for that town, Dr. F. E. Wynne, made a striking speech in which he detailed some of the factors that had caused the infant mortality of Sheffield to decrease during the past two years. "Personally," said Dr. Wynne, "I believe that every water tap that replaces a standpipe in the yard, every tramcar that takes people away from the centre of the town, every drain and sewer constructed, every highway paved and cleansed, every insanitary backyard paved, and every privy midden converted to the water carriage system, is a nobler monument to public health progress than the handsomest sanatorium or the best equipped clinic."

WHILE so much has lately been demanded in the better education of nurses, it is natural that the desire for the better education of hospital officers should once again be in request. Mr. W. H. Harper, secretary of the Wolverhampton General Hospital, made a useful suggestion the other day when addressing the Midlands Branch of the Association of Hospital Officers. "It would" (he said) "be very beneficial to us all to arrange for a course of lectures in such subjects as architecture and building construction, accounting and hospital appeals, heating and hot-water installation, electrical work, &c. In many of these subjects our knowledge is far too superficial. We are living in a superficial age." We cordially endorse this suggestion, but everything will depend upon the type of lecturer. It is true that a general knowledge of the principles of building accounts, applied electricity, should be part of a general education. But these, we think, should not occupy too much time in the proposed course. That which is really needed is a set of lectures by men, not in the habit of lecturing, who have had to solve the problems considered themselves. Such men are hard to find, but if they could be induced, in the form of an account of their own experiences, when placing contracts, for instance, to make a present of the result of their experiences, the lectures would be very instructive. A man of hospital experience, who can speak, and be persuaded to give the time required, would be a priceless addition to the ordinary "course of lectures." Most of us learn these things, in so far as we learn them, in the course of an administrative work. One who has so learned, and is able to present his knowledge, would be worth the finding.

THE first Annual Report on the Health of Blackburn by Dr. Allen Daley, the Medical Officer of Health, is a very readable and interesting publication, which should be a powerful means of stimulating local public interest in matters of health. Dr. Daley is fully alive to the value of health propaganda work, and writes as follows in his report: —

The efforts of Public Health Authorities have in the past been directed mainly to the removal of gross forms of insanitation by, for example, the provision of a pure water supply, efficient scavenging, and housing schemes. This work of creating an improved sanitary environment is still proceeding, but a perusal of any report on the health of the inhabitants of a large town will demonstrate that an essential need in a scheme for improving health is the enlightenment of the people. They must be taught to live healthy lives so that they can resist disease: they must know how disease in its early stages can be recognised and how the spread of infectious disease can be avoided. Expenditure on health education will be amply repaid by a saving in the cost of treatment of persons who have become ill through ignorance of how to keep well. No sanitary problem was ever solved only by treating those who fall ill. We must begin at the beginning and endeavour to prevent healthy people from be coming ill. The Chief Medical Officer of the Ministry of Health puts it well when he says:

> "An essential part of any national health policy is the instruction in the principles and practice of hygiene of the great mass of the people. In this as in other spheres of human affairs ignorance is the chief curse. We are only now, as knowledge grows, becoming aware of the immeasurable part played by ignorance in the realm of disease. It is hardly too much to say that in proportion as knowledge spreads in a population, disease and incapacity decline, and this becomes more evident as the gross forms of pandemic disease are overcome. As in the individual so in the community, knowledge is the sheet anchor of preventive medicine—knowledge of the way of health, knowledge of the causes and channels of disease, knowledge of remedy."

It has, therefore, been decided to form in Blackburn a Committee for the purpose of educating public opinion on health matters. This Committee will carry out its work by various methods: Press notices containing in plain language essential points regarding the prevention of ill-health; popular lectures illustrated by cinema films or lantern slides will be given, and travelling exhibitions relating, for example, to tuberculosis or child welfare will be invited to visit the town. A strong Committee on these lines was formed in December, 1920, and excellent work has already been done.

SOUTHPORT Infirmary authorities are intending to revise their rules, in order to give workers more direct representation on the board of management. Until recently, the governors have elected one or two worker managers, but the arrangement has not been altogether successful.

It is now proposed to make provision that any trade union, society, workshop, &c., subscribing ten guineas annually, shall be allowed to nominate one governor for each ten guineas so subscribed. These wage-earner governors are to form a separate panel for the purpose of electing eight members of the board of management, and these managers will be additional to the managers elected at the annual court of governors.

The wage-earner governors will be entitled to all the privileges of other governors, save that they will not vote in the election of the board of management apart from the wage-earner members. In addition, the wage-earner governors are to be called together every three months, so that the worker representatives on the management will be kept in touch with the feelings of the worker subscribers.

